# Risk factors associated with progression from pre‐eclampsia to eclampsia: A prospective cohort study and population‐wide data analysis

**DOI:** 10.1111/aogs.15154

**Published:** 2025-05-14

**Authors:** Annettee Nakimuli, Brittany A. Jasper, Sarah Nakubulwa, Moses Adroma, Jackline Akello, Imelda Namagembe, Musa Sekikubo, Eve Nakabembe, Ashley Moffett, Catherine E. Aiken

**Affiliations:** ^1^ Department of Obstetrics and Gynaecology Makerere University and Mulago National Referral Hospital Kampala Uganda; ^2^ Department of Obstetrics and Gynaecology University of Cambridge, The Rosie Hospital and NIHR Cambridge Biomedical Research Centre Cambridge UK; ^3^ Department of Pathology and Centre for Trophoblast Research University of Cambridge Cambridge UK

**Keywords:** low‐resource setting, maternal age, mortality, obstetrics, pregnancy, risk factors, socioeconomic status

## Abstract

**Introduction:**

Eclampsia is a life‐threatening complication of pre‐eclampsia. There are currently no means of reliably identifying women with pre‐eclampsia who are at the highest risk of progression to eclampsia and would thus benefit from prioritization for intensive monitoring and urgent delivery. This is particularly challenging in obstetric settings where resources are limited. We identify risk factors for the progression of pre‐eclampsia to eclampsia in low‐ and middle‐income settings.

**Material and methods:**

Women diagnosed with pre‐eclampsia were prospectively recruited at a single tertiary referral centre in urban Uganda (2011–2016). Multivariable logistic regression models were used to identify risk factors that predicted the likelihood of progression to eclampsia. Key findings were validated in a geographically, socioeconomically, and ethnically distinct population using population‐wide hospital admission data from Ecuador (2021–2023).

**Results:**

In urban Uganda, progression from pre‐eclampsia to eclampsia was associated with nulliparity (OR 2.4; 95% CI: 1.1–5.4, *p* = 0.03), Baganda ethnicity (OR 1.9; 95% CI: 1.1–3.9, *p* = 0.01), unskilled/unemployed paternal occupation (OR 2.8; 95% CI: 1.3–6.4, *p* = 0.03), and a trend toward younger maternal age (OR 0.9; 95% CI: 0.9–1.0 per year; *p* = 0.09). Risk of progression to eclampsia was not related to the severity of pre‐eclampsia or the number of antenatal clinic visits. In Ecuador, population‐wide analysis showed that progression to eclampsia was associated with younger maternal age (*p* < 0.001) and a trend toward public vs privately funded obstetric care (*p* = 0.09).

**Conclusions:**

Eclampsia risk extends beyond clinical markers of pre‐eclampsia severity, with socioeconomic factors and maternal age playing crucial roles in disease progression. A targeted, context‐specific approach prioritizing high‐risk young women with socioeconomic vulnerabilities could optimize healthcare resources and mitigate severe hypertensive disorder risks.

## Graphical abstract

Younger maternal age and lower socioeconomic status emerged as key risk factors for progression from pre‐eclampsia to eclampsia in two low‐ and middle‐income countries (Uganda and Ecuador), challenging the traditional focus on disease severity alone. This suggests a need to broaden risk assessment criteria.

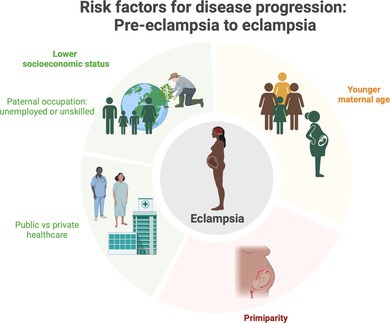

**Abbreviations**
CIs: confidence intervals
HIV: human immunodeficiency virus
LMICs: low‐income and middle‐income countries
SGA: small‐for‐gestational age

**Key message**

Lower socioeconomic status, nulliparity, and younger maternal age are significant predictors of eclampsia progression in low‐middle income settings, challenging disease severity as the main risk indicator. These findings underscore the necessity of comprehensive, contextualized risk assessment strategies that integrate social determinants of health.

## 1 INTRODUCTION

Pre‐eclampsia remains a leading cause of maternal mortality worldwide, affecting 2–8% of pregnancies globally.^1^ Sub‐Saharan Africa bears a disproportionate burden of pre‐eclampsia‐related maternal deaths.^2^, ^3^, ^4^ Over 99% of pre‐eclampsia‐related maternal deaths occur in low‐income and middle‐income countries (LMICs), with only 1% occurring in high‐income countries.^2^ Despite this, there is a lack of evidence on predicting and preventing severe complications of pre‐eclampsia in LMICs, particularly eclampsia. In resource‐limited obstetric settings, where clinical demand often outstrips resources, it is vital that women at the highest risk of severe adverse outcomes are prioritized. The ability to predict the progression of pre‐eclampsia to eclampsia would therefore be a valuable clinical tool.

Severe pre‐eclampsia can lead to maternal mortality through multiple mechanisms, many of which are thought to be primarily driven by placental dysfunction and endothelial dysfunction, ultimately leading to end‐organ damage.^7^, ^8^ The risk of maternal death is considerably increased in the presence of eclampsia when compared to pre‐eclampsia alone.^7^, ^8^, ^9^, ^10^ However, the pathogenesis of eclampsia remains unclear. It has been suggested that women with pre‐eclampsia exhibit cerebrovascular changes similar to those seen in hypertensive encephalopathy, with compromised autoregulation of cerebral blood flow.^10^ This disruption in autoregulation during acute hypertension results in increased hydrostatic pressure, reduced cerebral vascular resistance, and a resultant increased permeability of the blood–brain barrier, thereby changing the neural microenvironment.^10^, ^11^ These alterations in cerebral blood flow, coupled with hypercoagulability, inflammation, and neuro‐excitability, may precipitate cerebral oedema, micro‐bleeds, and neuronal damage, ultimately manifesting as an eclamptic seizure.^1^, ^11^

Maternal death following eclamptic seizures may occur due to aspiration pneumonia, cerebral hemorrhage, and oedema, or cardiac arrest.^7^, ^10^ However, the precise mechanisms underlying the progression from pre‐eclampsia to eclampsia remain poorly understood.^4^, ^7^, ^10^, ^12^

We aimed to utilize a large, well‐characterized, prospectively collected dataset of cases and controls from a low‐resource setting in urban Uganda and population‐wide cohort data from a middle‐resource setting in Ecuador to elucidate the incidence and risk factors associated with the progression to eclampsia in the context of pre‐eclampsia.

## 2 MATERIAL AND METHODS

### 2.1 Ugandan study population

A prospective cohort study was conducted at Mulago National Referral and Teaching Hospital in Kampala, Uganda. Mulago Hospital is the largest maternity center in Sub‐Saharan Africa and the tertiary referral center for Uganda. With an annual delivery rate of 31,000 to 33,000 births, it is one of the busiest maternity hospitals in the world.

### 2.2 Data collection

Participants were actively recruited in three waves: July 2009, August 2010 to June 2011, and September 2014 to December 2016. Sensitivity analyses were performed demonstrating that the results remain substantively unchanged across years or waves of recruitment. Women diagnosed with pre‐eclampsia by their clinical care provider were consecutively recruited from the maternity unit during each recruitment phase. Data were collected at the initial presentation by research midwives using a structured researcher‐administered questionnaire, supplemented by information abstracted from medical records.

### 2.3 Study design and outcome measures

The primary outcome was the occurrence of eclampsia, defined as generalized tonic–clonic seizure activity in the presence of diagnosed pre‐eclampsia, with no other identifiable cause for seizure activity, based on the clinical judgment of the treating clinicians.^10^ The pre‐eclampsia status of every recruited woman was verified against diagnostic criteria, which were based on a context‐appropriate adaptation of the 2013 ACOG criteria.^13^ Pre‐eclampsia was defined as systolic blood pressure ≥140 mmHg and/or diastolic blood pressure ≥90 mmHg on at least two occasions 4 h apart, accompanied by either ≥1 proteinuria on a urine dipstick or clinical seizure activity. Due to resource constraints typical of low‐resource settings,^14^ routine laboratory blood testing was not available for all women presenting with hypertension and proteinuria, limiting the full application of the ACOG criteria.^13^ Women with known chronic hypertension, epilepsy, or renal disease were excluded. The severity of pre‐eclampsia was determined by the maximum recorded systolic and diastolic blood pressures during the delivery episode and the highest level of proteinuria detected on a urine dipstick.

Maternal, paternal, and fetal characteristics were collected through structured patient interviews with research midwives and review of medical records. Maternal characteristics included age (in years) and ethnicity. All women included in the study were Black African. Ethnicity in mothers was self‐reported and categorized into the local Indigenous group (Baganda) or other ethnic groups. Parity and gravidity information was recorded and subsequently used in modeling as a factor that reflected whether the woman was nulliparous or parous. Women were asked about severe febrile illnesses during pregnancy, likely representing malaria given local prevalence and population susceptibility.^14^ Data regarding maternal human immunodeficiency virus (HIV) status were also obtained from laboratory records. The number of antenatal clinic visits recorded at Mulago National Referral and Teaching Hospital was collected.

Fetal birth weight (kg) was recorded by the midwife at the time of delivery. Birth weights were converted to gestational age and sex‐adjusted centiles using an international reference standard.^15^ Small‐for‐gestational age (SGA) was defined as birth weight < 10th centile for gestational age and sex. Gestational age (weeks) was calculated based on either last menstrual period or ultrasound scan. The analytic sample comprised singleton infants born between 28 and 43 weeks estimated gestation. Additional participant characteristics included maternal education, employment, and marital status. Maternal education was categorized into three levels that reflected the highest level of education received; “ordinary”: education up to 12 years, “advanced”: education up to 16 years, and “higher”: education or any formal training leading to a qualification beyond 16 years. Maternal occupation was obtained by research midwives asking women in their own words what their job entailed and then classifying their occupation using the ISCO‐08 system^16^ and collapsed into categories: “professional,” “skilled,” or “unemployed/unskilled.” Similarly, paternal occupation was reported by women on behalf of their partners and categorized in the same way as for maternal occupation.

The primary outcome was eclampsia, which was diagnosed by the clinical team caring for each study participant. Neurological imaging (CT/MR) was not routinely performed on all women with clinical seizure activity to exclude other diagnoses. Such imaging was based on clinical indication and available resources. We also examined the risk of adverse fetal and neonatal outcomes, including prematurity, mode of delivery, and perinatal death in women who experienced eclampsia.

### 2.4 Ecuador study population

Anonymised data for all births complicated by pre‐eclampsia from 2021 to 2023 was obtained from a national registry of hospital discharges compiled by the Ecuadorian National Institute of Statistics and Census and the Ecuadorian Ministry of Health. Cases were classified by three‐character ICD‐10 codes: O14 for preeclampsia and O15 for eclampsia. Other variables extracted were maternal age, ethnicity, funding of care, and place of care. Maternal age was treated as a numeric variable. Ethnicity was grouped into Mestiza (mixed), Andean, Afro‐Ecuadorean, and other ethnicities, and was self‐reported by patients.

### 2.5 Statistical analyses

Descriptive statistics were used to summarize the characteristics of the study populations; n (%) or mean (SD) as appropriate. Comparisons between groups were performed using either Student's *t*‐test or the Mann–Whitney *U*‐test for continuous variables, depending on data normality. Categorical variables were compared using Pearson's chi‐squared test or Fisher's exact test, as appropriate.

Logistic regression analysis was utilized to identify possible risk factors for adverse outcomes, including eclampsia, and results expressed as adjusted odds ratios with 95% confidence intervals (CIs). Co‐variates were retained in models if they were either significantly associated with eclampsia risk in univariate analysis, biologically plausibly related to the outcome, or improved the model fit, as evaluated by the Akaike information criterion. The area under the receiver operating characteristic curve was used to evaluate the predictive value of our models for risk of progression to eclampsia. The relationship between maternal age and eclampsia risk was evaluated using a generalized additive model, fit using the R package “mgcv.”

Power calculations were conducted using Monte Carlo methods to determine the likelihood of detecting significant associations between various factors and the development of eclampsia. A p‐value of <0.05 was considered significant for all tests. Data analyses were performed using R statistical software version 3.5.1.

## 3 RESULTS

### 3.1 Progression from pre‐eclampsia to eclampsia in urban Uganda

The overall rate of progression to eclampsia in women with pre‐eclampsia was 5.9% (63/1075). In unadjusted analysis (Table 1), factors associated with increased risk of eclampsia included younger maternal age (*p* < 0.001) and nulliparity (*p* < 0.001). Women who were unmarried (*p* = 0.008) or whose partners were in unskilled or no employment (*p* = 0.04) were also at higher risk of eclampsia (Table 1). Women who experienced eclampsia were more likely to be of local Baganda ethnicity (*p* = 0.05). Eclampsia risk in this study population was not influenced by antenatal clinic attendance, HIV status, or febrile illness during pregnancy (Table 1).

**TABLE 1:** Comparison of maternal, paternal, and fetal characteristics between women with pre‐eclampsia who did and did not develop eclampsia in Uganda.

Characteristic	No eclampsia *N* = 1012	Eclampsia *N* = 63	*p* value
Highest systolic blood pressure (mmHg)	172.5 (±21.6)	174.9 (±22.1)	0.40
Highest diastolic blood pressure (mmHg)	115.5 (±15.7)	117.7 (±16.6)	0.30
Proteinuria			
2+	364 (35.9%)	16 (25.4%)	0.20
3+	376 (37.2%)	29 (46.0%)	
4+	272 (26.9%)	18 (28.6%)	
Maternal age (years)			
<25	517 (51.1%)	49 (77.8%)	<0.001***
25–29	269 (26.6%)	6 (9.5%)	
≥30	226 (22.3%)	8 (12.7%)	
Fetal sex			
Female	526 (52.9%)	26 (41.3%)	0.07
Male	469 (47.1%)	37 (58.7%)	
Gestational age at delivery	36.7 (±3.0)	37.3 (±3.5)	0.14
Nulliparous			
Yes	512 (50.9%)	16 (25.4%)	<0.001***
No	493 (49.1%)	47 (74.6%)	
Visits to antenatal clinic			
0–2	225 (22.3%)	11 (17.5%)	0.21
3–4	454 (44.9%)	39 (61.9%)	
≥5	90 (8.9%)	5 (7.9%)	
Unknown	242 (23.9%)	8 (12.7%)	
Maternal ethnicity			
Baganda	609 (60.2%)	47 (74.6%)	0.02*
Other	403 (39.8%)	16 (25.4%)	
Maternal employment			
Unemployed/unskilled	378 (37.4%)	26 (41.3%)	0.47
Skilled/clerical	438 (43.2%)	24 (38.1%)	
Professional/managerial	167 (16.5%)	7 (11.1%)	
Unknown	29 (2.9%)	6 (9.5%)	
Paternal employment			
Unemployed/unskilled	175 (17.3%)	16 (25.4%)	0.04*
Skilled/clerical	288 (28.5%)	25 (38.7%)	
Professional/managerial	291 (28.8%)	11 (17.5%)	
Unknown	258 (25.5%)	11 (17.5%)	
Maternal education			
Ordinary	359 (35.5%)	23 (36.5%)	0.67
Higher	112 (11.1%)	5 (7.9%)	
Advanced	69 (6.8%)	3 (4.8%)	
Unknown	474 (46.6%)	32 (50.8%)	
Marital status			
Unmarried	122 (12.1%)	17 (27.0%)	0.008**
Married	647 (63.9%)	38 (60.3%)	
Unknown	243 (24.0%)	8 (12.7%)	
HIV status			
Positive	49 (4.8%)	3 (4.8%)	0.95
Negative	943 (93.2%)	60 (95.2%)	
Unknown	20 (2.0%)	0 (0%)	
Febrile illness during pregnancy			
Yes	198 (19.6%)	9 (14.3%)	0.13
No	575 (56.8%)	46 (73%)	
Unknown	239 (23.6%)	8 (12.7%)	

*Note*: *p < 0.05; **p < 0.01; ***p < 0.001.

In adjusted analysis, women who were having their first baby (*p* = 0.03) and Baganda women (*p* = 0.01) had an approximately twofold increased risk of eclampsia compared to other women with pre‐eclampsia (Table 2). Women whose partners were in unskilled or no employment were also at higher risk (*p* = 0.03, Table 2). Younger women had a greater risk of eclampsia, although this effect no longer met the threshold for statistical significance (Table 2). There was no difference in eclampsia risk between women delivering a male vs female fetus. The best predictive model for eclampsia in women with pre‐eclampsia demonstrated reasonable but not high predictive power, with an area under the curve of 0.73.

**TABLE 2:** Multivariable analysis of factors that may be associated with the disease progression from pre‐eclampsia to eclampsia in Uganda.

Characteristic	Odds ratio	95% CI	*p* value
Nulliparous			
No	Ref		
Yes	2.4	1.1–5.4	0.03*
Ethnicity			
Other	Ref		
Baganda	1.9	1.1–3.9	0.01*
Fetal sex			
Male	1.6	0.9–2.9	0.11
Female	Ref		
Paternal employment			
None/unskilled	2.8	1.3–6.4	0.03*
Skilled/clerical	2.2	1.1–4.7	0.06
Professional/managerial	Ref		
Maternal age (per year)	0.9	0.9–1.0	0.09

*Note*: *p < 0.05; **p < 0.01; ***p < 0.001.

### 3.2 Neonatal outcomes in Uganda

**FIGURE 1:**
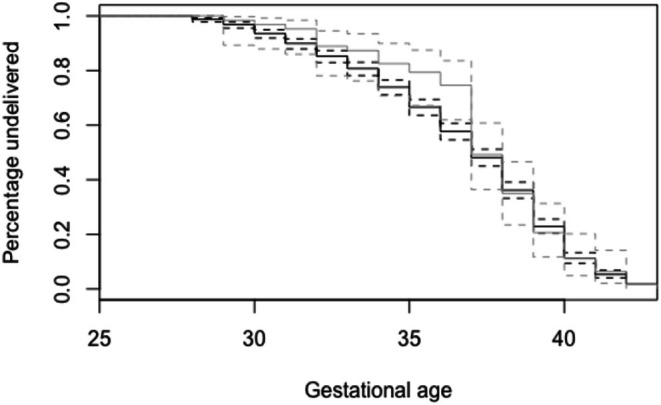
Gestational age at delivery in women with pre‐eclampsia (dark gray line) and eclampsia (light gray line). Dashed lines represent 95% confidence intervals for each line.

**FIGURE 2:**
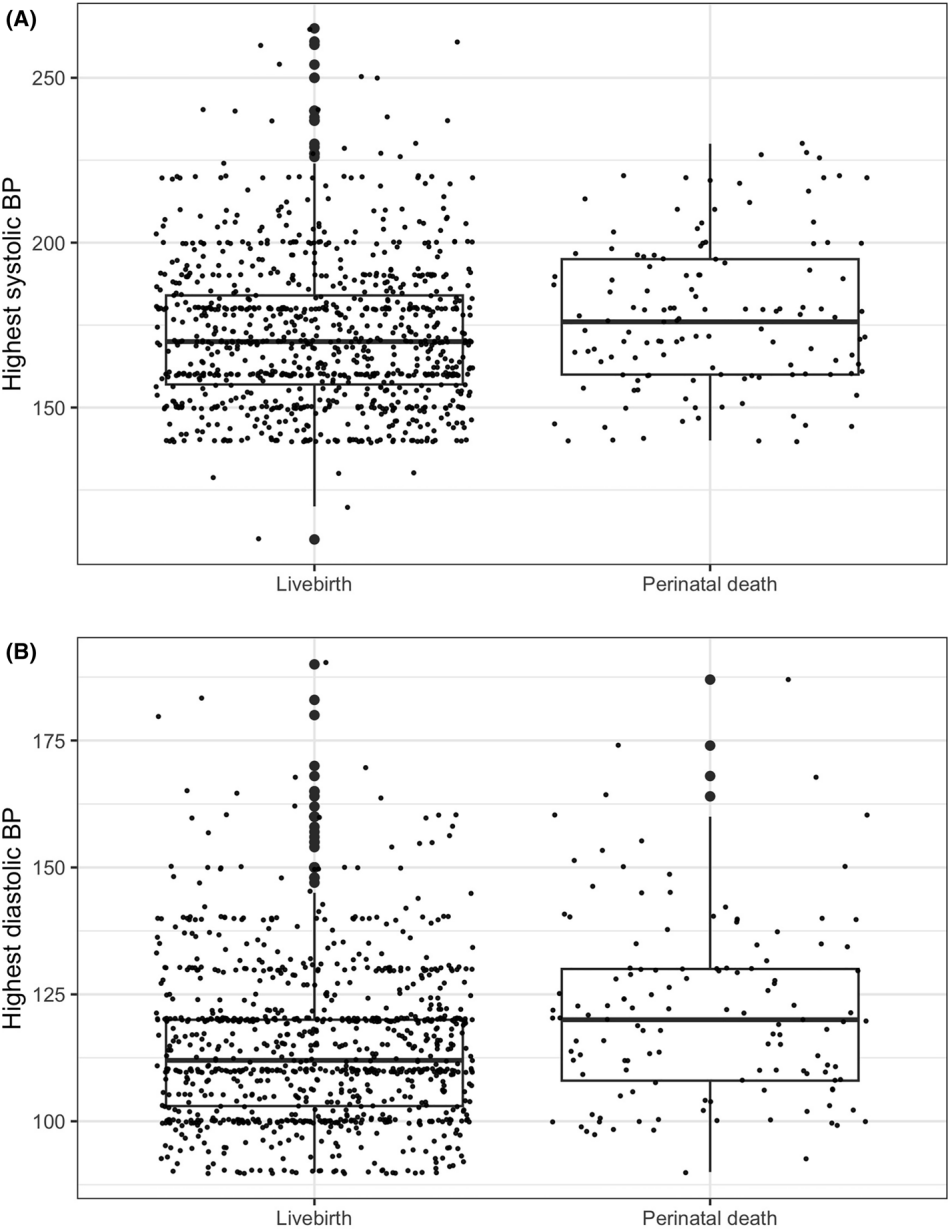
Boxplot showing (A) systolic blood pressure *p* < 0.01) and (B) diastolic blood pressure (*p* < 0.001) in livebirths vs perinatal deaths.

Women whose disease progressed to eclampsia delivered at slightly later gestations (36.7 ± 3wks v. 37.3 ± 3.5wks; Table 1) and had lower rates of both very preterm and moderate‐to‐late preterm delivery (*p* = 0.03; Table 3) than those with pre‐eclampsia (Figure 1). There was no difference in the likelihood of being delivered by either induced vaginal delivery or Cesarean section (Table 3). There was no difference in the rates of perinatal death between women with pre‐eclampsia and eclampsia in unadjusted analysis (Table 3). Although there was no association between the severity of pre‐eclampsia and risk of eclampsia, perinatal death was more common in women with higher blood pressures (Figure 2). In adjusted analyses, these associations were unaltered. Co‐variates that influenced eclampsia risk (paternal employment status, maternal ethnicity, nulliparity, and maternal age) did not significantly influence the risk of perinatal death within the context of pre‐eclampsia.

**TABLE 3:** Comparison of maternal and fetal outcomes between women with pre‐eclampsia who did and did not develop eclampsia in Uganda.

Characteristic	No eclampsia *N* = 1012	Eclampsia *N* = 63	*p* value
Birth weight (kg)	2.5 (±0.8)	2.6 (±0.7)	0.27
Birth weight centile	39.3 (±34.8)	38.1 (±34.2)	0.78
SGA (10th centile)			
No	655 (64.7%)	43 (68.2%)	
Yes	310 (30.6%)	19 (30.2%)	
Unknown	47 (4.6%)	1 (1.6%)	0.81
Prematurity			
≤32 weeks	149 (14.7%)	7 (11.1%)	
33–36 weeks	279 (27.6%)	9 (14.3%)	
≥37 weeks	584 (57.7%)	47 (74.6%)	0.03*
Mode of delivery			
Spontaneous vaginal	242 (23.9%)	10 (15.9%)	
Induced vaginal	317 (31.3%)	20 (31.7%)	
Cesarean section	442 (43.7%)	28 (44.4%)	
Unknown	11 (1.1%)	5 (8.0%)	0.48
Perinatal death			
Yes	894 (88.3%)	52 (82.5%)	
No	118 (11.7%)	11 (17.5%)	0.17

*Note*: *p < 0.05; **p < 0.01; ***p < 0.001.

### 3.3 Validation in a population‐based cohort in Ecuador

**FIGURE 3:**
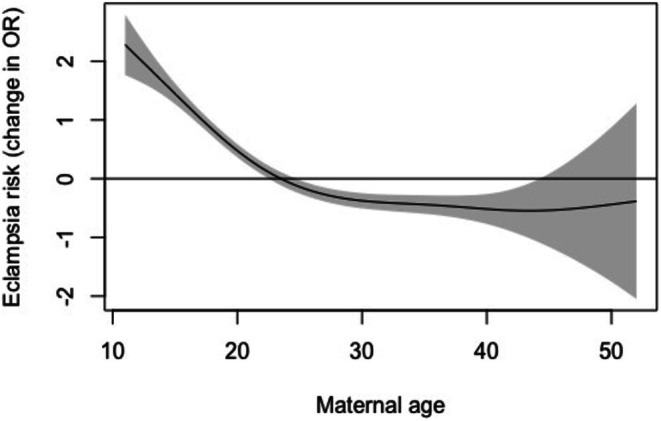
Relationship between maternal age and risk of eclampsia in women with pre‐eclampsia. Shaded areas represent 95% confidence intervals.

We sought to investigate our key finding that progression of pre‐eclampsia to eclampsia is more common in women who are younger and facing socioeconomic challenges in a different LMIC context. In Ecuador during 2021–2023, 3.4% of women with pre‐eclampsia went on to develop eclampsia (795/23537). The key factor associated with eclampsia risk was younger maternal age (*p* < 0.001; Table 4). There was a greater likelihood of women who experienced eclampsia being in public rather than privately funded care and in specialist care rather than general hospital or clinic settings, although these associations did not reach the threshold for statistical significance (Table 4). There was no impact of ethnicity on the risk of eclampsia in this population (Table 4). In adjusted analysis, maternal age and delivery in higher level care facilities were both significantly associated with eclampsia risk. The relationship between maternal age and pre‐eclampsia risk was non‐linear, with reducing risk with advancing age until ~30 years, and static risk thereafter (Figure 3).

**TABLE 4:** Comparison of maternal characteristics between women with pre‐eclampsia who did and did not develop eclampsia in population‐wide data from Ecuador.

Characteristic	No eclampsia *N* = 22 742	Eclampsia *N* = 795	*p* value
Maternal age (years)	27.1 (±7.2)	23.2 (±7.2)	<0.001***
Maternal ethnicity			
Mestiza (mixed)	19 746 (86.8%)	688 (86.5%)	0.465
Andean	1036 (4.6%)	44 (5.5%)	
Afro‐Ecuadorean	648 (2.8%)	18 (2.3%)	
Other	1312 (5.8%)	45 (5.7%)	
Place of care			
Local clinic	4109 (18.1%)	120 (15.1%)	0.09
District hospital	15 790 (69.4%)	569 (71.6%)	
Specialist hospital	2843 (12.5%)	106 (13.3%)	
Funding of care			
Public	21 357 (93.9%)	758 (95.3%)	0.09
Private	1385 (6.1%)	37 (4.7%)	

*Note*: *p < 0.05; **p < 0.01; ***p < 0.001.

## 4 DISCUSSION

Rates of severe complications^7^, ^8^, ^9^, ^10^ associated with pre‐eclampsia in urban Uganda were higher than those reported in other populations, both in LMIC and HIC settings.^7^, ^9^, ^17^, ^18^, ^19^ In the Ugandan study population, 5.9% of women with pre‐eclampsia progressed to eclampsia, compared to 3.4% in Ecuador, a middle‐income South American setting. Notably, 12% of pregnancies affected by pre‐eclampsia in Uganda resulted in perinatal death, which is higher than rates documented elsewhere.^8^, ^17^, ^18^, ^19^

Across both settings, the risk of eclampsia was highest in younger women and in those with socioeconomic challenges. In Ecuador, there was no increased risk of progression to eclampsia in women of Black African ancestry, but within the Ugandan population (all of whom were Black African), eclampsia was more prevalent among women from the Baganda group compared to other ethnic groups. In urban Uganda, we found that eclampsia risk was not associated with the severity of pre‐eclampsia, but there was a strong correlation between severity and the risk of perinatal death.

Our findings challenge the prevailing assumption that eclampsia risk is primarily driven by disease severity.^7^, ^9^, ^20^ However, mothers who progressed to eclampsia were delivered later than those who experienced pre‐eclampsia only. It is possible that younger women, those having their first baby, or those with fewer resources present later in the course of their disease for care. Although the severity of pre‐eclampsia did not correlate to the risk of developing eclampsia, the overall time spent at risk may increase the possibility of seizure activity. The reasons for later delivery in younger women or those having their first baby may also reflect decreased risk perception on the part of obstetricians or reluctance to intervene with operative deliveries in younger women. Although all the women in our Ugandan cohort (a low‐resource setting) and the majority in Ecuador (a middle‐resource setting) were cared for within the public system without the expectation that they would have to pay for their clinical care, the likelihood of presenting for emergency care may still be heavily influenced by socioeconomic factors. Pre‐eclampsia can develop rapidly,^7^, ^8^, ^9^, ^10^ and it may be that women with more financial resources have fewer barriers to accessing urgent care, for example, transport, family support, work, or other responsibilities. Interestingly, there was no correlation in the Ugandan cohort between eclampsia risk and either planned antenatal clinic visits or maternal education, suggesting that while access to longer term monitoring or information about recognizing pre‐eclampsia may reduce the incidence of pre‐eclampsia,^21^ it is not protective specifically against progression to eclampsia in this context.

In other populations, pre‐eclampsia is well‐documented as being more common in nulliparous women.^7^, ^10^ However, the progression to eclampsia in these women is less frequently reported, likely due to the rarity of eclampsia.^9^ In our Ugandan data, where both maternal age and nulli were reported, nulliparity was a stronger influence than maternal age on eclampsia risk. A potential explanation is that women who develop eclampsia are at a higher risk of severe disability or death,^7^, ^8^, ^9^, ^10^, ^11^ which may prevent them from experiencing subsequent pregnancies or make them more likely to avoid future pregnancies due to trauma or fear of adverse outcomes. However, in Uganda, where high parity is often culturally valued and contraceptive use is low, this explanation may be less applicable.^22^, ^23^, ^24^ Another possible explanation is that women, whether they have previously experienced pre‐eclampsia or not, may be more skilled at recognizing warning signs of complications and seeking medical attention promptly, rather than attributing them to normal pregnancy symptoms. This timely response to medical evaluation and treatment may reduce the risk of progression to eclampsia.

Our study raises several important questions that warrant further investigation. In particular, the link between paternal socioeconomic status and maternal eclampsia risk is not straightforward to untangle. Paternal employment in a professional or managerial role likely reflects some aspect of higher socioeconomic status, but is not attributable to a direct effect of access to antenatal clinical care or maternal education. Higher socioeconomic status may confer advantages that might help mitigate the risk of progression to eclampsia, such as better dietary choices, overall health status, or other factors not observable in this population. This is an important area for future study with a more comprehensive set of markers for different dimensions of socioeconomic status.

One limitation of our study is the absence of measured body mass index in the study populations, as it is not routinely performed. This limits our ability to assess obesity as a potential risk factor for disease progression to eclampsia.^7^, ^10^ Gestational age was often estimated based on the last menstrual period rather than a first‐trimester crown‐rump length measurement, as is common in low‐resource obstetric settings and constitutes a source of potential heterogeneity within the cohort.^14^

Our study also has some important strengths. A key positive aspect is the use of a large, well‐characterized dataset from a low‐resource urban setting in Uganda where pre‐eclampsia rates are high, and maternal mortality rates exceed WHO targets.^4^, ^5^, ^6^ The study population also has a high rate of eclampsia, which is highly unusual in other populations and hence provides a unique opportunity to study risk factors that are not often easily examined, especially in low‐resource settings. A further key strength is the addition of further analysis in a population‐wide dataset from another LMIC in Ecuador. Although this data lacks granularity, it allows interrogation of key factors including maternal age and socioeconomic markers.

## 5 CONCLUSION

Eclampsia risk, in contrast to perinatal death risk, is influenced by factors beyond disease severity, including socioeconomic factors and maternal age. These results highlight the need for a stratified approach prioritizing high‐risk women through community health worker screenings, focusing on those who are young, nulliparous, and/or with socioeconomic vulnerabilities. Approaches that may be effective include mobile health technologies and targeted training for local midwives, which can enable cost‐effective risk monitoring, while subsidized nutritional support and simplified screening checklists can extend healthcare reach. By implementing these context‐specific strategies, healthcare systems can optimize scarce resources and significantly reduce severe hypertensive disorder risks in pregnancy.

## AUTHOR CONTRIBUTIONS

Annettee Nakimuli: conceptualization, formal analysis, writing—original draft, supervision, funding acquisition; Brittany A. Jasper: methodology, data curation, formal analysis, writing—review and editing; Sarah Nakubulwa: investigation, writing—review and editing; Moses Adroma: investigation, writing—review and editing; Jackline Akello: investigation, writing—review and editing; Imelda Namagembe: investigation, writing—review and editing; Musa Sekikubo: investigation, writing—review and editing; Eve Nakabembe: investigation, writing—review and editing; Ashley Moffett: conceptualization, writing—review and editing; Catherine E. Aiken: formal analysis, writing—original draft.

## FUNDING INFORMATION

This work was funded by the Wellcome Trust (094073/Z/10/B), and a Wellcome Trust Uganda Postdoctoral Fellowship in Infection and Immunity held by AN, funded by a Wellcome Trust Strategic Award, grant number 084344. Supported by NURTURE fellowship to AN, grant number D43TW010132. This work was also supported through the DELTAS Africa Initiative (grant number 107743/Z/15/Z). The DELTAS Africa Initiative is an independent funding scheme of the African Academy of Sciences (AAS)'s Alliance for Accelerating Excellence in Science in Africa (AESA) and supported by the New Partnership for Africa's Development Planning and Coordinating Agency (NEPAD Agency) with funding from the Wellcome Trust (grant number 107743/Z/15/Z) and the UK government. We also wish to acknowledge the support of the Wellcome Trust—Cambridge Centre for Global Health Research Cambridge—Africa Programme. CEA is supported by the NIHR Cambridge Biomedical Research Centre. The views expressed are those of the authors and not necessarily those of the NIHR or the Department of Health and Social Care. The funders had no role in the data collection, analysis, and interpretation of data; in the writing of the report; or in the decision to submit the article for publication.

## CONFLICT OF INTEREST STATEMENT

The authors have no conflicts of interest or competing financial interests to declare.

## ETHICS STATEMENT

This study was approved by Makerere University's Faculty of Medicine Research and Ethics Committee (Reference numbers 2009‐083 and 2014‐065) on May 12, 2009 and May 5, 2014 respectively. All study participants gave informed consent. Ecuadorean data are entirely anonymized and freely available online (see data availability statement); therefore, no institutional IRB was required for this aspect.

## Data Availability

The raw data from the Ugandan study are not publicly available, due to the nature of the consent given by participants. However, information, including the full study protocol, data dictionary, and anonymised raw data extracts, is available to researchers under an appropriate Data Transfer Agreement. Enquiries should be sent to the first author (annettee.nakimuli@mak.ac.ug). The Ecuadorean dataset is freely available online from the Ecuadorean Ministry of Health website: https://www.ecuadorencifras.gob.ec/estadisticas/.
